# Temporal trends in pulse pressure and mean arterial pressure in Chinese children and adolescents over two decades (1991–2015)

**DOI:** 10.3389/fcvm.2022.910810

**Published:** 2022-09-09

**Authors:** Xinyue Zhang, Yunying Zhu, Shuting Li, Xinxin Ye, Leying Hou, Yating You, Chenyu Wang, Yuhang Wu, Junmeng Zhang, Yinlin Wang, Peige Song, Xi Mao

**Affiliations:** ^1^School of Public Health and Women's Hospital, Zhejiang University School of Medicine, Zhejiang University, Hangzhou, China; ^2^Stomatological Hospital, Zhejiang University School of Medicine, Zhejiang University, Hangzhou, China; ^3^Zhejiang University School of Stomatology and Key Laboratory of Oral Biomedical Research of Zhejiang Province, Zhejiang University School of Medicine, Zhejiang University, Hangzhou, China; ^4^Institute of Health and Wellbeing, University of Glasgow, Glasgow, United Kingdom; ^5^Zhejiang University-University of Edinburgh Institute, Zhejiang University School of Medicine, Zhejiang University, Hangzhou, China; ^6^Jiangxi Province Key Laboratory of Preventive Medicine, School of Public Health, Nanchang University, Nanchang, China; ^7^Bloomberg School of Public Health, Johns Hopkins University, Baltimore, MD, United States; ^8^Institute of Cartography and Geographic Information System, Chinese Academy of Surveying and Mapping, Beijing, China

**Keywords:** pulse pressure, mean arterial pressure, China, children, adolescents, trends

## Abstract

**Background:**

Pulse pressure (PP) and mean arterial pressure (MAP) are well-established markers of cardiovascular risk. In this study, we aimed to assess the temporal trend and associated factors of PP and MAP in Chinese children and adolescents.

**Methods:**

From the China Health and Nutrition Survey 1991–2015, a total of 11,123 children and adolescents aged 7–17 years were included. Stratified analyses and generalized estimating equation (GEE) were conducted to compare the trends of PP and MAP by age and sex over two decades, along with the calculation of average relative increase (ARI). Moreover, multivariable linear regression was used to estimate the associated factors of PP and MAP.

**Results:**

During 1991–2015, upward trends were observed in both PP and MAP levels, with ARI of 0.30 and 0.34%, respectively. PP was higher in boys [PP_1991_ 33.9 mmHg (*95%CI*, 33.40–34.33) to PP_2015_ 35.4 mmHg (34.74–36.15)] than in girls [PP_1991_ 33.3 mmHg (32.83–33.72) to PP_2015_ 34.3 mmHg (33.59–34.99)]. PP was also higher in participants aged 13–17 years [PP_1991_ 36.1 mmHg (35.63–36.62) to PP_2015_ 38.3 mmHg (37.35–39.21)] than in those aged 7–12 years [PP_1991_ 31.5 mmHg (31.09–31.88) to PP_2015_ 33.7 mmHg (33.16–34.30)]. Similar results were found in MAP. Participants with high economic status, general obesity and central obesity, were more likely to have wider PP (β_*higheconomicstatus*_ = 0.60, *95% CI*, 0.19–1.02; β_*generalobesity*_ = 1.38, 0.87–1.89; β_*centralobesity*_ = 1.34, 0.70–1.97; all *P-values* < 0.001) and higher MAP (β_*higheconomicstatus*_ = 0.82, 0.38–1.26; β_*generalobesity*_ = 2.88, 2.33–3.42; β_*centralobesity*_ = 3.14, 2.47–3.80; all *P-values* < 0.001). Body mass index (BMI) and waist circumference (WC) were positively correlated with PP (β_*BMI*_ = 0.18, 0.13–0.24; β_*WC*_ = 0.10, 0.08–0.12; both *P-values* < 0.001) and MAP (β_*BMI*_ = 0.43, 0.37–0.49; β_*WC*_ = 0.20, 0.18–0.22; both *P-values* < 0.001). In addition, rural setting and glucose level were positively associated with PP (both *P* < 0.05), while north region residency, uric acid, and total cholesterol were found to be positively associated with MAP (all *P* < 0.05).

**Conclusion:**

PP and MAP levels have been increasing dramatically in Chinese children and adolescents over the last two decades. Age, sex, economic status, geographic factors, anthropometric and cardiometabolic factor were positively associated with PP and MAP in pediatric population.

## Introduction

Cardiovascular disease (CVD) is a group of conditions that include heart failure, heart attack, stroke, coronary heart disease, and many other diseases around heart vessels (1). According to the Global Burden of Disease Study 2015, CVD has surpassed cancer, respiratory disease and diabetes mellitus, becoming the leading cause of non-communicable disease death, putting an enormous strain on public health and healthcare systems (2). From 1990 to 2019, the global trend for disability-adjusted life years (DALYs) and years of life lost due to CVD grew dramatically, with DALYs doubling from 17.7 to 34.4 million (3). The American Heart Association indicates that there is merely 0.2% population in China possesses ideal cardiovascular health (4). Currently, modifiable CVD-related risk factors, such as elevated blood pressure, general obesity, high fasting plasma glucose, and dyslipidaemia are becoming increasingly common in children and adolescents (5, 6). Several longitudinal studies suggest that people with higher blood pressure levels in childhood are more likely to experience CVDs and metabolic syndrome in adulthood (7, 8).

Pulse Pressure (PP) is the difference between systolic blood pressure (SBP) and diastolic blood pressure (DBP), which is determined by significant artery stiffness and flow pulsatility (9). PP has been suggested to be useful in predicting CVD events in previous studies (10–12). Wide PP might illustrate that blood flow, aortic diameter, and wall stiffness are not matched when the aorta is surrounded by considerable remodeling stress in children (13). Since arterial stiffness is a determinant of PP and a predictor of hypertension, increased PP in children may reflect pathological vascular remodeling and precipitate further adverse vascular remodeling and incident hypertension (11). Mean Arterial Pressure (MAP) is a weighted average of SBP and DBP, which is determined by small resistance artery function and cardiac output (9). Pathophysiological evidence from recent studies showed that wide PP and high MAP are precursors of isolated systolic hypertension (14). Thus, it is critical to pay attention to PP and MAP in children and adolescents.

Previous studies have widely revealed the temporal changes of pediatric blood pressure across the globe (15, 16), whereas the trends of PP and MAP in children and adolescents are still under-researched, especially in China. To fill this knowledge gap, we used the China Health and Nutrition Survey (CHNS), a nationwide longitudinal study, to assess the dynamic changes of PP and MAP in Chinese children and adolescents over two decades (1991–2015). In addition, we also explored the associations of demographic, geographic, anthropometric and cardiometabolic factors with PP and MAP on pediatric.

## Methods

### Study design and study population

CHNS is a nationwide longitudinal household-based study, which has been conducted every 2 or 4 years since 1989. CHNS data are currently available for the years of 1989, 1991, 1993, 1997, 2000, 2004, 2006, 2009, 2011, and 2015. A multistage random cluster process was used to draw the sample. First, counties and cities in covered provinces were divided into three groups (high-, middle-, low-income) according to economic status. Then, four counties (one low-, two middle-, and one high-income) and two cities (usually the provincial capital and a lower-income city) were selected randomly. Third, one community and three rural villages in each chosen county were randomly selected, as well as two communities and two suburban villages of each chosen city. In each community or village, all the members of 20 households were selected randomly. More details on survey procedures and the rationale of the CHNS could be found in the cohort profile (17). The lists and locations of the surveyed provinces are shown in Supplementary Table 1 and Supplementary Figure 1.

CHNS was approved by the Institutional Review Board of the University of North Carolina at Chapel Hill, the National Institute of Nutrition and Food Safety, China Centers for Disease Control and Prevention and the Ministry of Health Sino-Japanese Friendship Hospital. All respondents had signed the consent form.

### Data collection

Data on demographics and socioeconomics (e.g., age, sex, setting, region, and household income) were collected through questionnaires by trained interviewers. Anthropometric measurements were implemented according to the standardized protocols by the World Health Organization (WHO) (18). Blood pressure was measured three times with a 3–5 min interval at sitting and with at least 1 min between recordings. Vertical mercury column sphygmomanometer was used in each wave (1). On a calibrated beam scale, weight was measured to the nearest 0.1 kg without jackets. Height was measured to the nearest 0.1 cm without shoes using a portable stadiometer (19). Waist circumference (WC) was measured using non-elastic tape between the lowest rib and the iliac crest in a horizontal plane at a point midway. Hip circumference (HC) was measured at the level of the maximal gluteal protrusion (18).

Blood samples were collected in CHNS 2009 (17). Major biomarkers included uric acid (UA), hemoglobin (Hb), high-density lipoprotein cholesterol (HDL-C), low-density lipoprotein cholesterol (LDL-C), total cholesterol (TC), triglyceride (TG), glucose, estimated glomerular filtration rate (eGFR), and alanine aminotransferase (ALT).

### Definitions

All participants were separated into two age groups: 7–12 and 13–17 years old, and age were standardized to the pediatric age structure in 2015 (1). Household income per capita at each wave (inflated to 2015 equivalency) was first transformed to its natural logarithm and then stratified into low, middle and high groups by tercile (20). Setting was classified into urban and rural areas. Region was divided into North China (Beijing, Liaoning, Heilongjiang, Shandong, and Henan) and South China (Shanghai, Jiangsu, Hubei, Hunan, Guangxi, Guizhou, and Chongqing).

Body mass index (BMI) was calculated as weight divided by height squared (kg/m^2^), waist-to-height ratio (WHtR) was calculated as WC divided by height, and waist to hip ratio (WHR) was calculated as WC divided by HC (21). According to the Working Group on Obesity in China (17), overweight was defined as a BMI between 85th and 95th percentile of sex and age group, and general obesity was defined as a BMI ≥ 95th percentile of sex and age group. Central obesity was defined as WC > 90th percentile in each sex and age group, or a WHtR ≥ 0.5, or a WHR ≥ 0.9 for boys and a WHR ≥ 0.85 for girls. The estimated glomerular filtration rate (eGFR) was calculated with Schwartz “original” formula, as k^*^height (cm)/Scr (mg/dL), and k was 0.70 if boys ≥ 13 years, and 0.55 otherwise (22, 23).

PP was calculated as SBP minus DBP, and MAP was calculated as one-third of SBP added to two-thirds of DBP (24).

### Statistical analysis

The characteristics of included participants were described in the form of means ± standard deviations (SDs) for normally distributed continuous variables, medians and interquartile ranges (IQRs) for non-normally distributed continuous variables, with differences calculated using *t*-test or Wilcoxon test. Numbers with percentage (%) were used for categorical variables, with differences calculated using Chi-square test. Three sets of analyses were conducted:

1) Based on CHNS 1991–2015, the trends of PP and MAP by age and sex were estimated, along with the calculation of average annual increase (AAI) and average relative increase (ARI). Generalized estimating equation (GEE) was adopted since some participants might have repeated in multiple waves;
2) Based on CHNS 1993 to 2015, multivariable linear regression was used to explore demographic, geographic and socioeconomic factors that were associated with PP and MAP;
3) Based on CHNS 2009, where biomarker data were available, the associations of cardiometabolic factors with PP or MAP were also explored using multivariable linear regression.

All statistical analyses were conducted using Stata statistical software (version 14.0; Stata Corporation, College Station, TX, USA), and a *P* < 0.05 was considered statistically significant in two-sided tests.

## Results

### Selection of study participants

**Figure 1:**
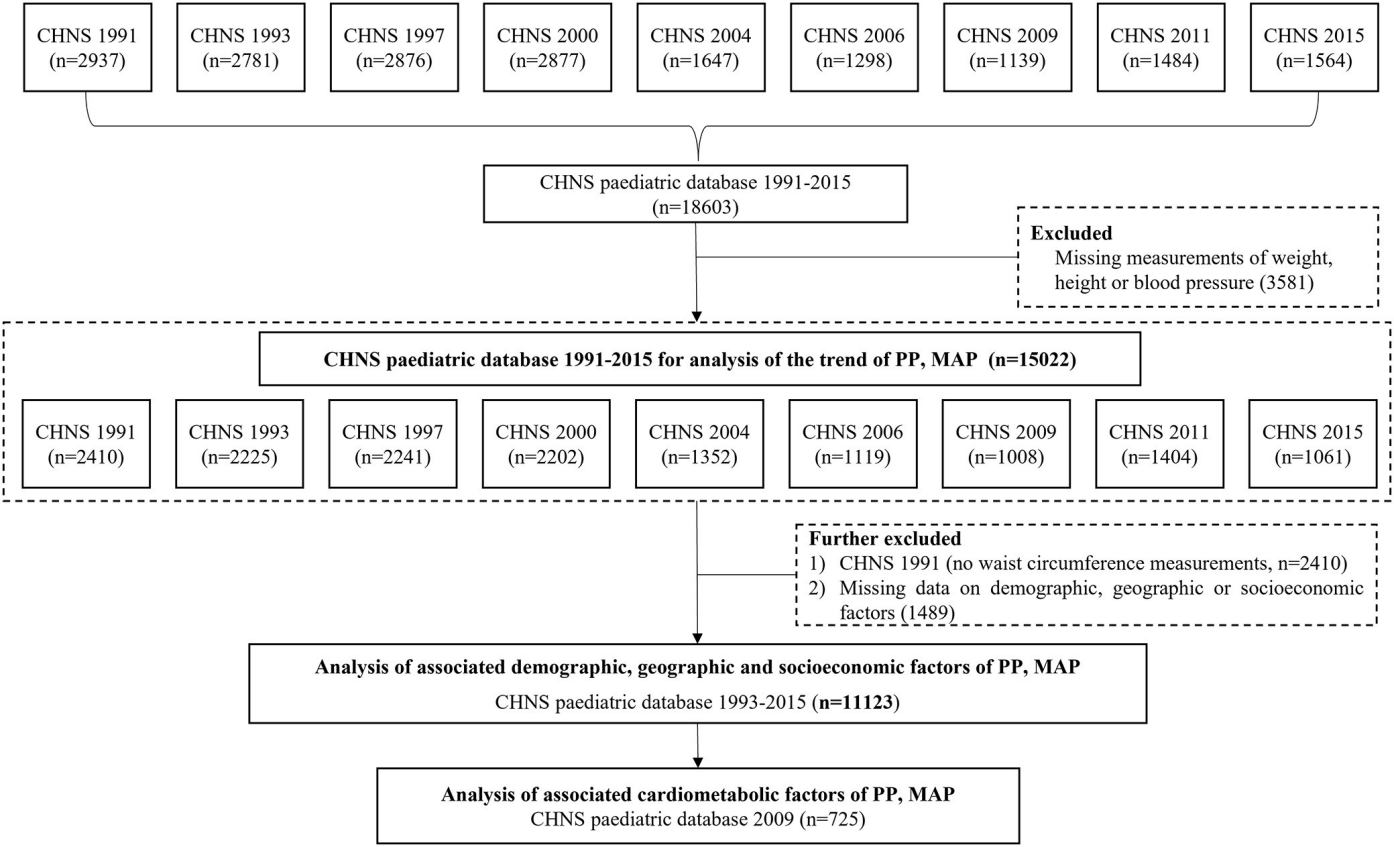
The flowchart for selecting subjects in CHNS 1991-2015 for the analysis of PP and MAP. Note: CHNS, the China Health and Nutrition Survey; PP, pulse pressure; MAP, mean arterial pressure.

From CHNS 1991 to 2015, a total of 15,022 records of children and adolescents (excluding those without weight, height, and BP measurements) were available for the trend analysis of PP and MAP. A total of 11,123 records from 1993 to 2015 had complete information on demography, geography, economic status, anthropometry, and thus were included in the analyses of associated factors. Additionally, a total of 725 records in 2009 wave were with data on biomarkers, and therefore were selected for the assessment of cardiometabolic factors (see Figure 1).

### Temporal trends in PP and MAP in children and adolescents

As outlined in Table 1 and Supplementary Figure 2, from 1991 to 2015, PP and MAP increased significantly (both *P-values* for trend < 0.001). Age-standardized mean PP increased from 33.6 mmHg (*95% CI*: 33.27–33.89) to 36.0 mmHg (*95% CI*: 35.48–36.55) while MAP increased from 73.8 mmHg (*95% CI*: 73.45–74.17) to 79.8 mmHg (*95% CI*: 79.21–80.44). PP was higher in boys [33.9 mmHg (*95%CI*, 33.40–34.33) in 1991 to 35.4 mmHg (*95%CI*, 34.74–36.15) in 2015] than in girls [33.3 mmHg (*95%CI*, 32.83–33.72) in 1991 to 34.3 mmHg (*95%CI*, 33.59–34.99) in 2015]. PP was also higher in those aged 13–17 years [36.1 mmHg (*95%CI*, 35.63–36.62) in 1991 to 38.3 mmHg (*95%CI*, 37.35–39.21) in 2015] than in those aged 7–12 years [31.5 mmHg (*95%CI*, 31.09–31.88) in 1991 to 33.7 mmHg (*95%CI*, 33.16–34.30) in 2015]. Similar results were found in MAP. During the same period, mean PP values increased across age and sex groups, with AAI of 0.04 mmHg in girls, 0.07 mmHg in boys, 0.09 mmHg in those aged 7–12 years, 0.09 mmHg in those aged 13–17 years and with ARI of 0.13% in girls, 0.19% in boys, 0.25% in those aged 7–12 years, 0.30% in those aged 13–17 years. This increasing trend is similar in MAP across age and sex groups.

**Table 1:** Trend of PP and MAP in Chinese children and adolescents, CHNS 1991–2015.

**Variable**	**1991 (8,604)**	**1993 (8,203)**	**1997 (8,592)**	**2000 (9,473)**	**2004 (9,209)**	**2006 (9,165)**	**2009 (9,499)**	**2011 (12,542)**	**2015 (11,296)**	**AAI (mmHg)**	**ARI (%)**	***P*** for trend^*^
**PP (mmHg), mean (95% CI)**												
Overall-crude	33.6	33.1	33.5	34.3	34.9	33.3	33.2	34.8	34.9	0.05	0.16	<0.001
	(33.26, 33.90)	(32.73, 33.41)	(33.19, 33.88)	(33.95, 34.70)	(34.39, 35.37)	(32.75, 33.75)	(32.67, 33.74)	(34.32, 35.18)	(34.39, 35.38)			
Overall-standardized	33.6	33.3	33.9	34.3	34.5	33.4	33.4	35.2	36.0	0.10	0.30	<0.001
	(33.27, 33.89)	(32.94, 33.60)	(33.49, 34.20)	(33.91, 34.70)	(33.98, 34.93)	(32.95, 33.92)	(32.91, 33.96)	(34.73, 35.62)	(35.48, 36.55)			
**Age group**												
7–12 years	31.5	31.3	31.9	32.6	32.2	31.5	31.8	33.3	33.7	0.09	0.30	<0.001
	(31.09, 31.88)	(30.91, 31.74)	(31.49, 32.31)	(32.18, 33.10)	(31.56, 32.87)	(30.87, 32.09)	(31.09, 32.47)	(32.78, 33.80)	(33.16, 34.30)			
13–17 years	36.1	35.4	36.0	36.3	37.3	35.7	35.4	37.2	38.3	0.09	0.25	<0.001
	(35.63, 36.62)	(34.84, 35.89)	(35.44, 36.57)	(35.74, 36.92)	(36.61, 37.96)	(34.89, 36.47)	(34.64, 36.23)	(36.44, 37.89)	(37.35, 39.21)			
**Sex**												
Boy	33.9	33.2	33.6	34.7	35.4	33.6	33.5	34.9	35.4	0.07	0.19	<0.001
	(33.40, 34.33)	(32.72, 33.70)	(33.14, 34.11)	(34.20, 35.29)	(34.64, 36.08)	(32.90, 34.31)	(32.79, 34.30)	(34.25, 35.49)	(34.74, 36.15)			
Girl	33.3	32.9	33.4	33.9	34.4	32.9	32.8	34.6	34.3	0.04	0.13	<0.001
	(32.83, 33.72)	(32.46, 33.37)	(32.94, 33.92)	(33.34, 34.35)	(33.70, 35.00)	(32.15, 33.56)	(32.04, 33.52)	(34.03, 35.22)	(33.59, 34.99)			
**MAP (mmHg), mean (95% CI)**												
Overall-crude	73.8	74.3	74.9	76.6	78.2	75.8	77.7	77.0	78.3	0.18	0.25	<0.001
	(73.41, 74.24)	(73.82, 74.69)	(74.44, 75.29)	(76.18, 77)	(77.69, 78.76)	(75.27, 76.39)	(77.07, 78.28)	(76.45, 77.45)	(77.66, 78.83)			
Overall-standardized	73.8	74.6	75.7	76.5	77.6	76.2	78.3	77.8	79.8	0.25	0.34	<0.001
	(73.45, 74.17)	(74.22, 75.00)	(75.28, 76.03)	(76.15, 76.92)	(77.09, 78.08)	(75.73, 76.70)	(77.67, 78.90)	(77.36, 78.26)	(79.21, 80.44)			
**Age group**												
7–12 years	69.5	70.6	71.4	73.4	73.8	72.0	75.3	74.0	76.7	0.30	0.43	<0.001
	(69.00, 69.97)	(70.08, 71.17)	(70.91, 71.93)	(72.84, 73.87)	(73.05, 74.57)	(71.36, 72.66)	(74.54, 76.08)	(73.45, 74.64)	(76.07, 77.38)			
13–17 years	79.1	79.0	80.1	80.5	82.2	81.1	81.4	81.7	82.7	0.15	0.19	<0.001
	(78.52, 79.67)	(78.46, 79.61)	(79.48, 80.66)	(79.90, 81.02)	(81.59, 82.84)	(80.33, 81.83)	(80.53, 82.21)	(81.02, 82.46)	(81.64, 83.81)			
**Sex**												
Boy	74.0	74.5	74.8	77.1	78.8	76.3	77.8	78.0	79.2	0.22	0.30	<0.001
	(73.37, 74.55)	(73.84, 75.10)	(74.24, 75.38)	(76.54, 77.69)	(78.05, 79.54)	(75.51, 77.11)	(77.00, 78.64)	(77.26, 78.72)	(78.39, 80.08)			
Girl	73.7	74.0	74.9	76.0	77.6	75.3	77.5	75.9	77.2	0.15	0.20	<0.001
	(73.08, 74.27)	(73.42, 74.61)	(74.29, 75.55)	(75.42, 76.57)	(76.83, 78.35)	(74.52, 76.05)	(76.61, 78.38)	(75.21, 76.55)	(76.40, 77.99)			

AAI (mmHg), average annual increase, was computed by dividing the difference of blood pressure between 1991 and 2015 by the number of years covered; ARI (%), Average relative increase was calculated by dividing the average annual change by the baseline mean level of PP or MAP in 1991.

* Age-adjusted trends from 1993 to 2011 were assessed by the generalized-estimating-equation method.

### Associations of demographic, geographic, socioeconomic and cardiometabolic factors with PP and MAP

Basic characteristics of the 11,123 included subjects were shown in Table 2. The mean age of participants was 11.86 years, and 5,851 (52.60%) participants were boys. The proportions of participants aged 7–12 years (57.30%), those living in rural areas (69.59%), those from South China (64.48%), and those with a high economic status (41.54%) were all slightly higher. Comparisons of demographic characteristics between the excluded and included subjects in CHNS 1991–2015 were shown in Supplementary Table 2. Besides, the basic characteristics of the 725 included subjects for the assessment of cardiometabolic factors were shown in Supplementary Table 3.

**Table 2:** Characteristics of included children in CHNS 1993–2015.

**Characteristic**	**1993–2015 combined (11,123)**	**1993 (1,236)**	**1997 (2,110)**	**2000 (2,117)**	**2004 (1,296)**	**2006 (1,062)**	**2009 (969)**	**2011 (1,350)**	**2015 (983)**
**Age, year**	11.86 ± 2.98	12.74 ± 3.14	11.66 ± 2.92	12.13 ± 2.73	12.43 ± 3.00	11.81 ± 3.10	11.58 ± 2.87	11.41 ± 3.03	10.72 ± 2.77
**Age group**									
7–12 years	6,373 (57.30)	543 (43.93)	1,281 (60.71)	1,148 (54.23)	617 (47.61)	618 (58.19)	592 (61.09)	839 (62.15)	735 (74.77)
13–17 years	4,750 (42.70)	693 (56.07)	829 (39.29)	969 (45.77)	679 (52.39)	444 (41.81)	377 (38.91)	511 (37.85)	248 (25.23)
**Sex**									
Boy	5,851 (52.60)	639 (51.70)	1,115 (52.84)	1,133 (53.52)	682 (52.62)	559 (52.64)	541 (55.83)	680 (50.37)	502 (51.07)
Girl	5,272 (47.40)	597 (48.30)	995 (47.16)	984 (46.48)	614 (47.38)	503 (47.36)	428 (44.17)	670 (49.63)	481 (48.93)
**Setting**									
Urban	3,383 (30.41)	386 (31.23)	647 (30.66)	579 (27.35)	370 (28.55)	310 (29.19)	264 (27.24)	508 (37.63)	319 (32.45)
Rural	7,740 (69.59)	850 (68.77)	1,463 (69.34)	1,538 (72.65)	926 (71.45)	752 (70.81)	705 (72.76)	842 (62.37)	664 (67.55)
**Region**									
North	3,951 (35.52)	286 (23.14)	743 (35.21)	916 (43.27)	527 (40.66)	410 (38.61)	363 (37.46)	432 (32.00)	274 (27.87)
South	7,172 (64.48)	950 (76.86)	1,367 (64.79)	1,201 (56.73)	769 (59.34)	652 (61.39)	606 (62.54)	918 (68.00)	709 (72.13)
**Economic status**									
Low	2,986 (26.85)	574 (46.44)	800 (37.91)	582 (27.49)	348 (26.85)	271 (25.52)	131 (13.52)	172 (12.74)	108 (10.99)
Middle	3,517 (31.62)	448 (36.25)	862 (40.85)	862 (40.72)	419 (32.33)	309 (29.10)	244 (25.18)	212 (15.70)	161 (16.38)
High	4,620 (41.54)	214 (17.31)	448 (21.23)	673 (31.79)	529 (40.82)	482 (45.39)	594 (61.30)	966 (71.56)	714 (72.63)
**Anthropometric measures**									
BMI, kg/m^2^	17.83 ± 3.37	17.79 ± 2.76	17.41 ± 2.8	17.66 ± 2.83	18.05 ± 2.87	17.72 ± 3.04	17.74 ± 3.26	18.36 ± 3.77	18.34 ± 5.73
WC, cm	62.86 ± 9.86	62.29 ± 8.46	61.41 ± 8.14	62.82 ± 8.31	63.99 ± 9.31	62.49 ± 9.93	63.08 ± 9.98	64.63 ± 12	63.04 ± 13.84
**General obesity**									
Normal	9,671 (86.95)	1,127 (91.18)	1,920 (91.00)	1,915 (90.46)	1,136 (87.65)	933 (87.85)	815 (84.11)	1,068 (79.11)	757 (77.01)
Overweight/obesity	1,452 (13.05)	109 (8.82)	190 (9.00)	202 (9.54)	160 (12.35)	129 (12.15)	154 (15.89)	282 (20.89)	226 (22.99)
**Central obesity**									
Normal	10,211 (91.80)	1,193 (96.52)	2,032 (96.30)	2,004 (94.66)	1,201 (92.67)	992 (93.41)	870 (89.78)	1,121 (83.04)	798 (81.18)
Central obesity	912 (8.20)	43 (3.48)	78 (3.70)	113 (5.34)	95 (7.33)	70 (6.59)	99 (10.22)	229 (16.96)	185 (18.82)

Data are given as mean ± SD or as number (proportion).

WC, waist circumference; BMI, body mass index.

As shown in Table 3, participants aged 13–17 years (vs. those aged 7–12 years) were more likely to have wider PP or higher MAP (β_*PP*_ = 0.75, *95% CI*: 0.69–0.80; β_*MAP*_ = 1.48, *95% CI*: 1.42–1.53, both *P-values* < 0.001). Compared with boys, girls were less likely to suffer from wider PP (β = −0.66, *95% CI*: −0.96 to −0.35, *P* < 0.001) or higher MAP (β = −0.92, *95% CI*: −1.24 to −0.60, *P* < 0.001). BMI and WC were significantly positively associated with PP (β_*BMI*_ = 0.18, *95% CI*: 0.13–0.24; β_*WC*_ = 0.10, *95% CI*: 0.08–0.12, both *P-values* < 0.001) and MAP (β_*BMI*_ = 0.43, *95% CI*: 0.37–0.49; β_*WC*_ = 0.20, *95% CI*: 0.18–0.22; both *P-values* < 0.001). In contrast with normal weight, general obesity and central obesity were both positively associated with wider PP (β_*generalobesity*_ = 1.38, *95% CI*: 0.87–1.89; β_*centralobesity*_ = 1.34, *95% CI*: 0.70–1.97, both *P-values* < 0.001) and higher MAP (β_*generalobesity*_ = 2.88, *95% CI*: 2.33–3.42; β_*centralobesity*_ = 3.14, *95% CI*: 2.47–3.80, both *P-values* < 0.001). In addition, those in high economics status were more likely to have wider PP (β = 0.60, *95% CI*: 0.19–1.02, *P* < 0.001) and higher MAP (β = 0.82, *95% CI*: 0.38–1.26, *P* < 0.001).

**Table 3:** The associations of demographic, geographic, and anthropometric factors and cardiometabolic factors with PP and MAP.

	**PP**		**MAP**	
	**β Coefficient (95% CI)**	***P*** **-value**	**β Coefficient (95% CI)**	***P*** **-value**
**Survey year**				
1993	0	Ref	0	Ref
1997	0.44 (−0.15, 1.02)	0.142	0.26 (−0.35, 0.88)	0.398
2000	0.91 (0.33, 1.50)	0.002	1.22 (0.60, 1.84)	<0.0001
2004	1.15 (0.50, 1.80)	0.001	2.25 (1.56, 2.93)	<0.0001
2006	−0.03 (−0.72, 0.66)	0.931	0.79 (0.07, 1.52)	0.032
2009	−0.09 (−0.80, 0.63)	0.81	2.77 (2.01, 3.52)	<0.0001
2011	1.55 (0.88, 2.22)	<0.0001	2.05 (1.34, 2.76)	<0.0001
2015	2.03 (1.30, 2.76)	<0.0001	4.10 (3.33, 4.87)	<0.0001
**Age (years)**	0.75 (0.69, 0.80)	<0.0001	1.48 (1.42, 1.53)	<0.0001
**Sex**				
Boy	0	Ref	0	Ref
Girl	−0.66 (−0.96, −0.35)	<0.0001	−0.92 (−1.24, −0.60)	<0.0001
**Setting**				
Urban	0	Ref	0	Ref
Rural	0.64 (0.30, 0.98)	<0.0001	0.09 (−0.27, 0.45)	0.618
**Region**				
North	0	Ref	0	Ref
South	−0.06 (−0.38, 0.27)	0.739	−1.49 (−1.83, −1.14)	<0.0001
**Economic status**				
Low	0	Ref	0	Ref
Middle	0.31 (−0.09, 0.71)	0.132	0.67 (0.24, 1.10)	0.002
High	0.60 (0.19, 1.02)	0.005	0.82 (0.38, 1.26)	<0.0001
**Obesity**				
Normal	0	Ref	0	Ref
Overweight/obesity	1.38 (0.87, 1.89)	<0.0001	2.88 (2.33, 3.42)	<0.0001
**Central obesity**				
Normal	0	Ref	0	Ref
Central obesity	1.34 (0.70, 1.97)	<0.0001	3.14 (2.47, 3.80)	<0.0001
**BMI, kg/m** ^2^	0.18 (0.13, 0.24)	<0.0001	0.43 (0.37, 0.49)	<0.0001
**WC, cm**	0.10 (0.08, 0.12)	<0.0001	0.20 (0.18, 0.22)	<0.0001
**CHNS 2009**				
**UA (μmol/L)**	0.01 (0.00, 0.02)	0.062	0.01 (0.00, 0.02)	0.04
**Hb (g/L)**	0.00 (−0.04, 0.04)	0.958	0.03 (−0.02, 0.07)	0.233
**HDL-C (mmol/L)**	−0.36 (−1.50, 0.78)	0.534	0.40 (−0.84, 1.65)	0.525
**LDL-C (mmol/L)**	−0.19 (−0.89, 0.52)	0.599	−0.48 (−1.25, 0.29)	0.218
**TC (mmol/L)**	0.17 (−0.01, 0.35)	0.058	0.21 (0.01, 0.40)	0.037
**TG (mmol/L)**	−0.54 (−1.43, 0.34)	0.228	0.32 (−0.64, 1.29)	0.514
**Glucose (mmol/L)**	0.88 (0.07, 1.70)	0.034	0.30 (−0.59, 1.19)	0.506
**eGFR (mL/min per 1.73 m** ^2^ **)**	0.04 (0.00, 0.09)	0.061	0.01 (−0.04, 0.06)	0.621
**ALT (U/L)**	−0.02 (−0.05, 0.01)	0.176	−0.02 (−0.05, 0.02)	0.315

Data are shown as β Coefficient (95% CI). Biomarkers were only measured in 2009.

WC, waist circumference; BMI, body mass index; UA, uric acid; Hb, hemoglobin; HDL-C, high-density lipoprotein cholesterol; LDL-C, low-density lipoprotein cholesterol; TC, total cholesterol; TG, triglyceride; eGFR, estimated glomerular filtration rate; ALT, alanine aminotransferase.

The association of geographical or cardiometabolic factors with PP or MAP was inconsistent. Participants living in rural areas (vs. urban areas) (β = 0.64, *95% CI*: 0.30–0.98, *P* < 0.001) or having increasing blood glucose (β = 0.88, *95% CI*: 0.07–1.70, *P* < 0.05) were more likely to have wider PP. Moreover, those living in north China (vs. south China) (β = 1.49, *95% CI*: 1.14–1.83, *P* < 0.001), having higher UA (β = 0.01, *95% CI*: 0.00–0.02, *P* < 0.05) or higher TC (β = 0.21, *95% CI*: 0.01–0.40, *P* < 0.05) were positively associated with higher MAP.

## Discussion

In this study, upward trends of both PP and MAP levels in Chinese children and adolescents were observed, with significant increases in PP (the difference from 1991 to 2015: 2.4 mmHg, ARI 0.30%) and MAP (the difference from 1991 to 2015: 6.0 mmHg, ARI 0.34%). PP and MAP levels were higher among boys than girls, and among participants aged from 13 to 17 years than those aged from 7 to 12 years, respectively. Besides, both central obesity and general obesity were strongly associated with higher PP and MAP in comparison to normal weight. In addition, participants living in rural or with higher fasting blood glucose were more likely to have a wider PP, while participants living in northern China, with higher UA or higher TC were more likely to have higher MAP.

To the best of our knowledge, this is the first study that reported the temporal trend and associated factors of PP and MAP levels among Chinese children and adolescents. Trends in PP and MAP over time in the US children were reported previously, and PP was found to be progressively higher with time, while MAP decreased (11). In contrast, our study showed that PP and MAP increased over the past 24 years in Chinese adolescents and children. The difference in MAP trends may be explained by the country's stage of economic development, as argued in previous studies of trends in blood pressure changes (25, 26). Theoretically, MAP is determined by small resistance artery function and cardiac output, and elevated MAP implies increased pressure on the heart and small vessels (9). The Framingham Heart Study pointed out that PP and MAP well explained the hemodynamics of altered arterial stiffness with impaired peripheral resistance (9). Our study adds to the important evidence of PP and MAP trends in children.

Age and sex were well-established determinants of elevated blood pressure in children and adolescents (27, 28). Our study showed that older children were more likely to have higher PP and MAP, which aligned with the fact that blood pressure increased with age along with the increased stiffness of blood vessels (28). As for sex, girls were less likely to have wider PP or higher MAP than boys in terms of numerical values, which was consistent with the findings in previous studies (11). It has been reported that multiple factors in the blood pressure regulation pathway differ by sex, including angiotensin-converting enzyme 2/Apelin signaling, sex hormone, endothelin-1, and sympathetic nervous (29). Besides, menstruation is a period of intense activity in girls and may alter the autonomic system due to intense hormonal production, leading to differences compared to boys (30).

We also observed strong associations of some physiological indicators with PP or MAP, including BMI, WC, UA, and TC. In our study, both central obesity and general obesity were positively associated with wider PP and higher MAP. Despite the pathogenesis of obesity directly influencing BP was pendent, there was still some widely held consensus. A case in point is that obesity may increase in the activity of the sympathetic nervous system, alterations in renal function and the renin-angiotensin-aldosterone system (31). Gruber et al. (32) stated that a profound remodeling in the hypothalamus occurs with elevated leptin levels and upregulation of a hypoxia-inducible factor 1a-vascular endothelial growth factor signaling axis in local astrocytes, which resulted in arterial hypertension. These researches in physiological mechanisms provide strong evidence for our results. UA and TC showed positive associations with MAP in our study. However, the relationship association between UA and PP was on the margin, which was partly due to the small sample size in 2009. Numerous observational data suggested a robust association between elevated serum uric acid (SUA) levels and an increased risk of incident hypertension in the whole population (33, 34). As for adolescents, mean SUA levels increased from 5.3 to 5.9 mg/dL with increasing blood pressure values from below the 80th to above the 90th percentile (35). Our findings were aligned with Soletsky's theory (36) that controlling uric acid can rectify prehypertension.

The influences of demographical factors on PP and MAP were also observed. Compared with participants in low economic status, participants with higher economic status were more likely to have wider PP and higher MAP. However, a prospective study in young Finns (37) reported the exact opposite conclusion: low family annual income in childhood predicts arterial stiffness, measured by elevated MAP. The contradictory result could be explained by the fact that China and Finland were in different nutrition transition stages as developed and developing countries (37). Moreover, in our results, children and adolescents in rural areas and north China tend to experience wider PP and higher MAP. Such association was also reported by a cross-sectional study in China, where relatively higher risks of high blood pressure (HBP), systolic HBP, and diastolic HBP were found in rural children (38).

## Limitations

Several limitations need to be considered. First, despite the large sample size and a wide variety of variables, the national representativeness of CHNS could not be well-guaranteed because of sampling imbalance. Second, anthropometric indicators were measured only in 2009, which shrank the scale of sample size to study the associations of such indicators with PP and MAP. Third, although valid associations were found between potential factors and PP or MAP levels, the multivariable linear regression of PP and MAP with potential factors cannot provide robust supporting evidence for further research. And given we have no relevant data, some other relevant factors that might have an impact on hemodynamics, like heart rate, were not included in the data analysis. However, considering the lack of attention on PP and MAP as potential CVD predictors, our study provides a solid first step for further explorations in this field. Fourth, dietary nutrition-related variables, such as carbohydrates, fat and salt intake (39), parental education and occupation (40) and the changes in pubertal/hormonal development also might affect the changes in PP and MAP levels (41, 42). However, due to lack of relevant data, we were unable to explore such relationships in Chinese children and adolescents over two decades.

## Conclusion

In summary, this study reported dramatically increasing PP and MAP levels in Chinese children and adolescents over the last two decades. Age, sex, economic status, geographic factors, anthropometric and cardiometabolic factors such as BMI, WC, UA were positively associated with PP and MAP in the Chinese pediatic population. PP and MAP may play an essential role in predicting CVD events and the diagnosis of hypertension. Our findings call for an emphasis on the application of PP and MAP for the early identification of vascular stiffness and elevated blood pressure in the younger generation.

## Data availability statement

The dataset presented in this study can be found in online repositories. Requests to access the datasets should be directed to https://www.cpc.unc.edu/projects/china.

## Ethics statement

The studies involving human participants were reviewed and approved by the National Institute for Nutrition and Health and Chinese Center for Disease Control and Prevention. Written informed consent was obtained from the participants. Written informed consent to participate in this study was provided by the participants' legal guardian/next of kin. Written informed consent was obtained from the individual(s), and minor(s)' legal guardian/next of kin, for the publication of any potentially identifiable images or data included in this article.

## Author contributions

XM and PS designed the study. XZ and YZ managed and analyzed the data and prepared the first draft. PS, XM, SL, XY, LH, YY, CW, YWu, JZ, and YWa critically revised the manuscript. All authors were involved in revising the paper and gave final approval of the submitted versions.

## Funding

This project was supported by the National Natural Science Foundation of China (Grant No. 72104211).

## Conflict of interest

The authors declare that the research was conducted in the absence of any commercial or financial relationships that could be construed as a potential conflict of interest.

## Publisher's note

All claims expressed in this article are solely those of the authors and do not necessarily represent those of their affiliated organizations, or those of the publisher, the editors and the reviewers. Any product that may be evaluated in this article, or claim that may be made by its manufacturer, is not guaranteed or endorsed by the publisher.
