# Supplementary material for: Temporal trends in pulse pressure and mean arterial pressure in Chinese children and adolescents over two decades (1991–2015)
Source: Front Cardiovasc Med. 2022 Sep 9;9:910810. doi: 10.3389/fcvm.2022.910810 (PMC9500211; doi:10.3389/fcvm.2022.910810)
Supplement: Supplementary file 1 [file Data_Sheet_1.pdf]

Title: Temporal Trends in Pulse Pressure and Mean Arterial Pressure in Chinese Children and Adolescents Over Two Decades (1991–2015)

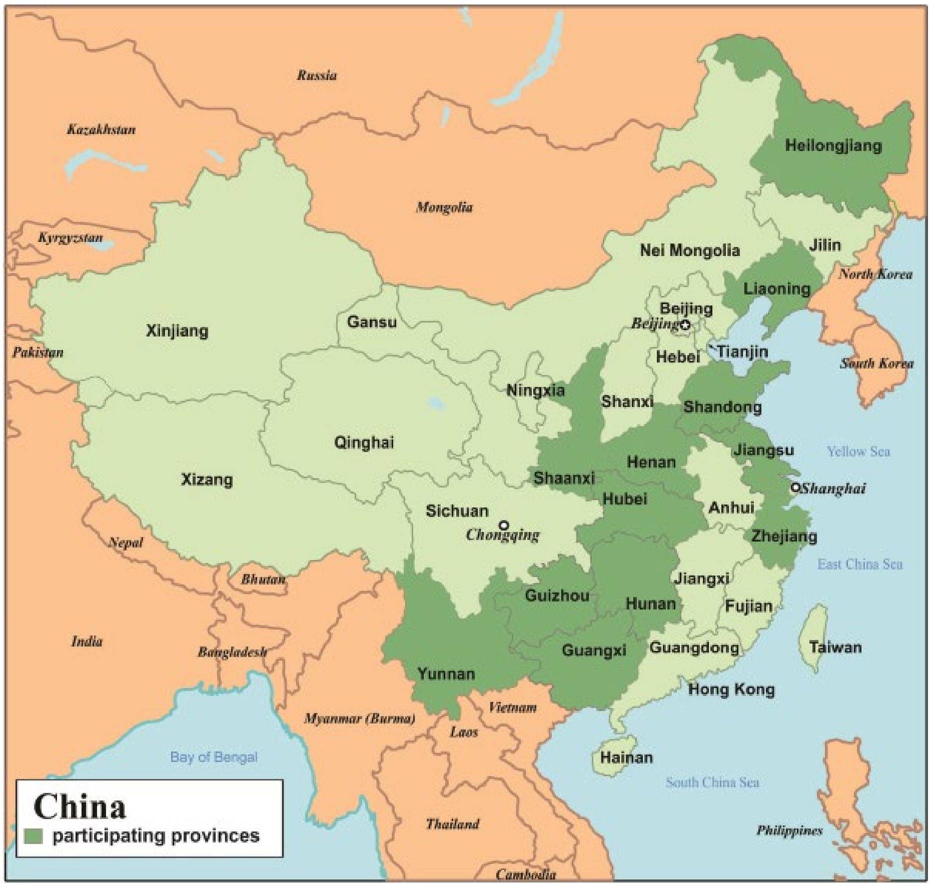

Supplementary Figure 1. Locations of investigated provinces in CHNS 1991-2015

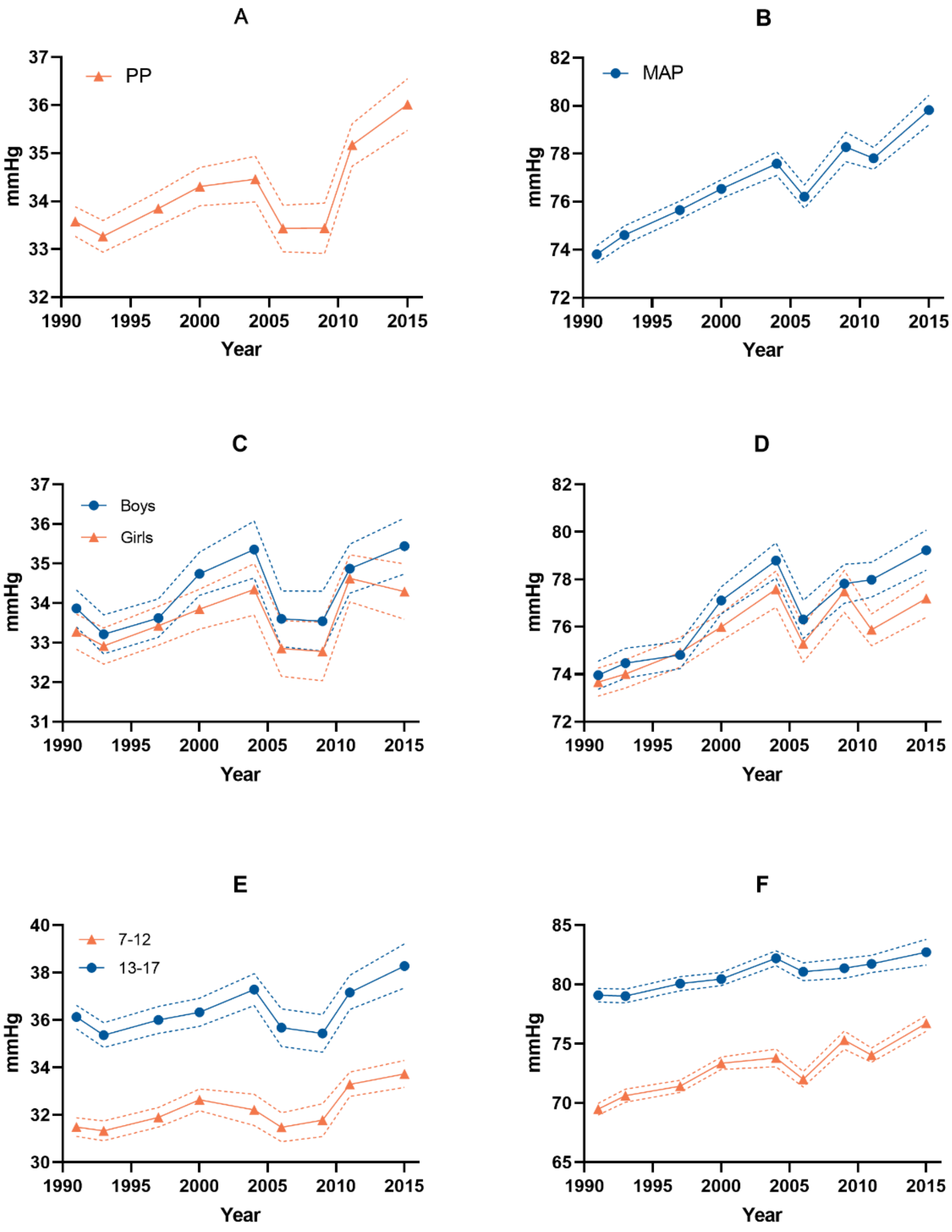

Supplementary Figure 2. Temporal trend in PP and MAP in Chinese children and adolescents over two decades

Note: *PP is pulse pressure and MAP is mean arterial pressure.
(A) Temporal trends in overall-standardized PP; (B) Temporal trends inoverall-standardized MAP.
(C) Temporal trends in PP stratified by gender; (D) Temporal trends in MAP stratified by gender; blue represents boys and orange represents girls.
(E) Temporal trends in PP stratified by age; (D) Temporal trends in MAP stratified by age; blue represents participants aged 13-17 years; orange represents participants 7-12 years.
Dashed lines indicate 95% confidence intervals.

Supplementary Table 1. Investigated provinces and in each wave of CHNS 1991-2015

Wave year	Investigated provinces
1991	Liaoning, Jiangsu, Shandong, Henan, Hubei, Hunan, Guangxi, Guizhou
1993	Liaoning, Jiangsu, Shandong, Henan, Hubei, Hunan, Guangxi, Guizhou
1997	Heilongjiang, Jiangsu, Shandong, Henan, Hubei, Hunan, Guangxi, Guizhou
2000	Liaoning, Heilongjiang, Jiangsu, Shandong, Henan, Hubei, Hunan, Guangxi, Guizhou
2004	Liaoning, Heilongjiang, Jiangsu, Shandong, Henan, Hubei, Hunan, Guangxi, Guizhou
2006	Liaoning, Heilongjiang, Jiangsu, Shandong, Henan, Hubei, Hunan, Guangxi, Guizhou
2009	Liaoning, Heilongjiang, Jiangsu, Shandong, Henan, Hubei, Hunan, Guangxi, Guizhou
2011	Beijing, Liaoning, Heilongjiang, Shanghai, Jiangsu, Shandong, Henan, Hubei, Hunan, Guangxi, Guizhou, Chongqing
2015	Beijing, Liaoning, Heilongjiang, Shanghai, Jiangsu, Shandong, Henan, Hubei, Hunan, Guangxi, Guizhou, Chongqing

Supplementary Table 2. Comparison of demographic characteristics between the excluded and included subjects in CHNS 1991-2015

Characteristics	1991-2015 combined			1991			1993			1997			2000		
	**Excluded (3,581)**	**Included (15022)**	**p value**	**Excluded (527)**	**Included (2,410)**	**p value**	**Excluded (556)**	**Included (2,225)**	**p value**	**Excluded (635)**	**Included (2,241)**	**p value**	**Excluded (675)**	**Included (2,202)**	**p value**
**Age, year**	12.58±3.42	11.81±2.99	<0.001	12.41±3.59	12.08±3.09	0.0342	12.35±3.56	11.82±3.1	0.0005	12.69±3.54	11.69±2.93	<0.001	13.65±2.99	12.12±2.74	<0.001
**Age group**															
7-12 years	1,604(44.79)	8,705(57.95)	<0.001	242(45.92)	1,322(54.85)	<0.001	266(47.84)	1265(56.85)	<0.001	280(44.09)	1349(60.20)	<0.001	215(31.85)	1,199(54.45)	<0.001
13-17 years	1,977(55.21)	6,317(42.05)		285(54.08)	1,088(45.15)		290(52.16)	960(43.15)		355(55.91)	892(39.80)		460(68.15)	1,003(45.55)	
**Sex**															
Male	1,910(53.34)	7,879(52.45)	0.339	287(54.46)	1,241(51.49)	0.217	273(49.1)	1,158(52.04)	0.214	338(53.23)	1,187(52.97)	0.907	351(52.00)	1,167(53.00)	0.650
Female	1,671(46.66)	7,143(47.55)		240(45.54)	1,169(48.51)		283(50.90)	1,067(47.96)		297(46.77)	1,054(47.03)		324(48.00)	1,035(47.00)	
**Setting**															
Urban	909(25.38)	4,304(28.65)	<0.001	120(22.77)	612(25.39)	0.207	117(21.04)	559(25.12)	0.045	147(23.00)	654(29.18)	0.003	167(24.74)	603(27.38)	0.175
Rural	2,672(74.62)	10,718(71.35)		407(77.23)	1,798(74.61)		439(78.96)	1,666(74.88)		488(77.00)	1,587(70.82)		508(75.26)	1,599(72.62)	

Characteristics	1991-2015 combined			1991			1993			1997			2000		
	**Excluded (3,581)**	**Included (15022)**	**p value**	**Excluded (527)**	**Included (2,410)**	**p value**	**Excluded (556)**	**Included (2,225)**	**p value**	**Excluded (635)**	**Included (2,241)**	**p value**	**Excluded (675)**	**Included (2,202)**	**p value**
**Region**															
North	1,493(41.69)	5,227(34.80)	<0.001	234(44.40)	720(29.88)	<0.001	243(43.71)	683(30.70)	<0.001	226(35.59)	771(34.40)	0.579	275(40.74)	955(43.37)	0.227
South	2,088(58.31)	9,795(65.20)		293(55.60)	1,690(70.12)		313(56.29)	1,542(69.00)		409(64.41)	1,470(65.60)		400(59.26)	1,247(56.63)	
**BMI, kg/m^2**	17.42±4.63	17.69±3.25	0.0395	15.4±1.86	17.38±2.78	<0.001	15.66±1.94	17.28±2.68	<0.001	15.66±2.05	17.43±2.78	<0.001	16.46±2.41	17.68±2.85	0.004
**Obesity**															
Normal	568(86.00)	13,228(88.00)	0.081	83(94.32)	2,222(92.20)	0.465	86(93.48)	2,046(91.96)	0.597	101(93.52)	2,042(91.12)	0.389	44(95.65)	1,983(90.05)	0.207
Overweight/ Obesity	94(14.00)	1,794(12.00)		5(5.68)	188(7.80)		6(6.52)	179(8.04)		7(6.48)	199(8.88)		2(4.35)	219(9.95)	
**Waist circumference, cm**	59.72±13.05	62.85±9.87	<0.001	NA	NA		58.28±8.45	62.33±8.46	<0.001	56.21±6.50	61.41±8.17	<0.001	60.01±8.78	62.76±8.34	0.002
**Central obesity**															
Normal	633(90.56)	10,505(91.75)	0.269	NA	NA		77(93.90)	1,206(96.48)	0.23	140(95.89)	2,066(96.27)	0.814	83(92.22)	2,057(94.75)	0.297

Characteristics	1991-2015 combined			1991			1993			1997			2000		
	**Excluded (3,581)**	**Included (15022)**	**p value**	**Excluded (527)**	**Included (2,410)**	**p value**	**Excluded (556)**	**Included (2,225)**	**p value**	**Excluded (635)**	**Included (2,241)**	**p value**	**Excluded (675)**	**Included (2,202)**	**p value**
Central obesity	66(9.44)	945(8.25)		NA	NA		5(6.10)	44(3.52)		6(4.11)	80(3.73)		7(7.78)	114(5.25)	

Continued

Characteristics	2004			2006			2009			2011			2015		
	**Excluded (295)**	**Included (1,352)**	**p value**	**Excluded (179)**	**Included (1,119)**	**p value**	**Excluded (131)**	**Included (1,008)**	**p value**	**Excluded (80)**	**Included (1,404)**	**p value**	**Excluded (503)**	**Included (1,061)**	**p value**
**Age, year**	11.73±3.21	12.46±3.00	0.0002	12±3.45	11.83±3.08	0.5013	12.28±3.17	11.58±2.87	0.009	12±3.41	11.39±3.04	0.0857	12.32±3.34	10.71±2.76	<0.001
**Age group**															
7-12 years	163(55.25)	641(47.41)	<0.001	93(51.96)	648(57.91)	<0.001	66(50.38)	615(61.01)	<0.001	42(52.50)	874(62.25)	<0.001	237(47.12)	792(74.65)	<0.001
13-17 years	132(44.75)	711(52.59)		86(48.04)	471(42.09)		65(49.62)	393(38.99)		38(47.50)	530(37.75)		266(52.88)	269(25.35)	
**Sex**															
Male	159(53.90)	715(52.88)	0.752	102(56.98)	592(52.90)	0.31	75(57.25)	559(55.46)	0.697	41(51.25)	713(50.78)	0.935	284(56.46)	547(51.56)	0.069
Female	136(46.10)	637(47.12)		77(43.02)	527(47.10)		56(42.75)	449(44.54)		39(48.75)	691(49.22)		219(43.54)	514(48.44)	
**Setting**															
Urban	65(22.03)	402(29.73)	0.008	50(27.93)	334(29.85)	0.602	36(27.48)	273(27.08)	0.923	34(42.50)	530(37.75)	0.395	173(34.39)	337(31.76)	0.3
Rural	230(77.97)	950(70.27)		129(72.07)	785(70.15)		95(72.52)	735(72.92)		46(57.50)	874(62.25)		330(65.61)	724(68.24)	

Characteristics	2004			2006			2009			2011			2015		
	**Excluded (295)**	**Included (1,352)**	**p value**	**Excluded (179)**	**Included (1,119)**	**p value**	**Excluded (131)**	**Included (1,008)**	**p value**	**Excluded (80)**	**Included (1,404)**	**p value**	**Excluded (503)**	**Included (1,061)**	**p value**
**Region**															
North	146(49.49)	548(40.53)	0.005	89(49.72)	433(38.70)	0.005	41(31.30)	374(37.10)	0.194	35(43.75)	442(31.48)	0.022	204(40.56)	301(28.37)	<0.001
South	149(50.51)	804(59.47)		90(50.28)	686(61.30)		90(68.70)	634(62.90)		45(56.25)	962(68.52)		299(59.44)	760(71.63)	
**BMI, kg/m^2**	16.54±2.71	18.1±2.92	<0.001	16.65±2.93	17.77±3.09	0.1085	16.96±4.28	17.79±3.35	0.4368	15.57±2.14	18.35±3.8	0.0208	21.12±6.52	18.28±5.6	<0.001
**Obesity**															
Normal	87(87.88)	1,181(87.35)	0.879	14(70.00)	979(87.49)	0.02	9(90.00)	845(83.83)	0.597	10(100.00)	1,111(79.13)	0.105	134(70.90)	819(77.19)	0.061
Overweight/ Obesity	12(12.12)	171(12.65)		6(30.00)	140(12.51)		1(10.00)	163(16.17)		0(0.00)	293(20.87)		55(29.10)	242(22.81)	
**Waist circumference, cm**	58.85±10.25	64.05±9.37	<0.001	59.91±11.15	62.51±9.91	0.058	66.35±12.07	63.07±9.95	0.192	55.57±7.21	64.49±11.96	0.068	63.14±20.15	63.03±13.72	0.927
**Central obesity**															

Characteristics	2004			2006			2009			2011			2015		
	**Excluded (295)**	**Included (1,352)**	**p value**	**Excluded (179)**	**Included (1,119)**	**p value**	**Excluded (131)**	**Included (1,008)**	**p value**	**Excluded (80)**	**Included (1,404)**	**p value**	**Excluded (503)**	**Included (1,061)**	**p value**
Normal	113(92.62)	1,235(92.37)	0.920	52(92.86)	1,028(93.54)	0.840	13(81.25)	895(89.68)	0.274	6(100.00)	1,168(83.31)	0.273	149(82.32)	850(81.18)	0.717
Central obesity	9(7.38)	102(7.63)		4(7.14)	71(6.46)		3(18.75)	103(10.32)		0(0.00)	234(16.69)		32(17.68)	197(18.82)	

Supplementary Table 3. Characteristics of included subjects in CHNS 2009.

	**Total (725)**	**Boys (398)**	**Girls (327)**
**Age (year)**	11.69±2.83	11.68±2.90	11.71±2.73
**Setting**			
Urban	197 (27.17)	88 (22.11)	109 (33.33)
Rural	528 (72.83)	310 (77.89)	218 (66.67)
**Region**			
North	260 (35.86)	144 (36.18)	116 (35.47)
South	465 (64.14)	254 (63.82)	211 (64.53)
**Economic status**			
Low	95 (13.10)	43 (10.80)	52 (15.90)
Middle	190 (26.21)	108 (27.14)	82 (25.08)
High	440 (60.69)	247 (62.06)	193 (59.02)
**BMI, kg/m^2**	17.69±3.23	17.72±3.31	17.65±3.12
**WC, cm**	63.13±9.55	64.01±10.12	62.05±8.71
**UA (μmol/L)**	303 (244-368)	321 (259-391)	286 (234-335)
**Hb (g/L)**	136 (128-146)	139 (130-149.5)	132 (126-141)
**HDL-C (mmol/L)**	1.43±0.56	1.42±0.35	1.45±0.73
**LDL-C (mmol/L)**	2.19±0.91	2.17±1.09	2.21±0.61
**TC (mmol/L)**	4.09±3.51	3.99±3.09	4.2±3.96
**TG (mmol/L)**	1.03±0.76	1.01±0.88	1.06±0.57
**Glucose(mmol/L)**	4.88±0.80	4.96±0.98	4.78±0.49
**eGFR (mL/min per 1.73 m^2)**	107.59±15.28	109.28±16.76	105.56±13.03
**ALT (U/L)**	13 (10-17)	14 (11-18)	12 (9-15)

Note: Data are given as mean±SD or median (IQR) or as number (proportion). Anthropometric factors were only measured in 2009. WC, waist circumference; BMI, body mass index; UA, uric acid; Hb, haemoglobin; HDL-C, high-density lipoprotein cholesterol; LDL-C, low-density lipoprotein cholesterol; TC, total cholesterol; TG, triglyceride; eGFR, estimated glomerular filtration rate; ALT, alanine aminotransferase
